# CircCRIM1 promotes nasopharyngeal carcinoma progression via the miR-34c-5p/FOSL1 axis

**DOI:** 10.1186/s40001-022-00667-2

**Published:** 2022-04-28

**Authors:** Weifeng He, Xiangqi Zhou, Yini Mao, YangJie Wu, Xiyang Tang, Sijia Yan, Sanyuan Tang

**Affiliations:** 1 Oncology Department, The Second People’s Hospital of Hunan Province, Changsha, 410007 Hunan People’s Republic of China; 2 Oncology Department, Affiliated Nanhua Hospital of University of South China, No. 336 Dong Feng South Road, Hengyang, 421002 Hunan People’s Republic of China; 3 Department of Neurosurgery, Xiangya Hospital, Central South University, Changsha, 410013 Hunan People’s Republic of China; 4 Oncology Department, The First Affiliated Hospital, University of South China, Hengyang, 421001 Hunan People’s Republic of China

**Keywords:** NPC, circCRIM1, miR-34c-5p, FOSL1

## Abstract

**Background:**

Nasopharyngeal carcinoma (NPC) is a rare malignancy with multiple risk factors (Epstein–Barr virus, etc.) that seriously threatens the health of people. CircRNAs are known to regulate the tumorigenesis of malignant tumours, including NPC. Moreover, circCRIM1 expression is reported to be upregulated in NPC. Nevertheless, the impact of circCRIM1 on NPC progression is not clear.

**Methods:**

An MTT assay was performed to assess cell viability. In addition, cell invasion and migration were assessed by the transwell assay. Dual luciferase assays were performed to assess the association among circCRIM1, miR-34c-5p and FOSL1. Moreover, RT-qPCR was applied to assess mRNA levels, and protein levels were determined by Western blot.

**Results:**

CircCRIM1 and FOSL1 were upregulated in NPC cells, while miR-34c-5p was downregulated. Knockdown of circCRIM1 significantly decreased the invasion, viability and migration of NPC cells. The miR-34c-5p inhibitor notably promoted the malignant behaviour of NPC cells, while miR-34c-5p mimics exerted the opposite effect. Moreover, circCRIM1 could bind with miR-34c-5p, and FOSL1 was identified to be downstream of miR-34c-5p. Furthermore, circCRIM1 downregulation notably inhibited the proliferation and invasion of NPC cells, while this phenomenon was significantly reversed by FOSL1 overexpression.

**Conclusion:**

Silencing circCRIM1 inhibited the tumorigenesis of NPC. Thus, circCRIM1 might be a novel target for NPC.

## Introduction

Nasopharyngeal carcinoma (NPC) is a malignant tumour that forms in the nasopharynx, occurring between the nose and the back of the throat [1]. While the incidence of NPC is high in some countries, especially in North Africa, Southeast Asia and southern China, it is not common among populations [2]. The symptoms of primary NPC include pain, trismus, otitis media and nasal regurgitation, which result from cranial nerve palsy (paralysis) and hearing loss [3]. Currently, surgery and radiotherapy are major strategies for the treatment of NPC, but the efficacies are still not ideal [4]. Moreover, the occurrence of NPC metastasis contributes to organ dysfunction [5]. Thus, new methods for NPC are urgently needed.

CircRNAs play important roles in cell biological processes, such as gene expression, posttranscriptional modification and protein synthesis [6, 7]. Circular RNAs (circRNAs) are a type of closed circular RNA molecule formed by reverse splicing, and their characteristics include high stability, biological evolutionary conservation and tissue expression specificity [8, 9]. Moreover, circRNA dysregulation is known to be closely related to NPC development. For instance, Huang et al. found that circNOTCH1 contributes to NPC progression by mediating the miR-34c-5p/c-Myc axis [10]; Pan et al. indicated that circLARP4 could alleviate NPC cell growth by repressing ROCK1 [11]. Moreover, circCRIM1 reportedly regulates the tumorigenesis of NPC by regulating FOXQ1 [12]. However, the role of circCRIM1 in NPC remains unclear.

MiRNAs (2–25 nts) are small RNAs that can modulate biological processes by regulating downstream mRNAs [13, 14]. Moreover, circRNAs are involved in NPC development by mediating miRNAs [15, 16]. For instance, the circRNA CDR1 aggravated NPC development by sponging miR-7-5p [15]. Furthermore, silencing circZNF609 suppressed NPC proliferation via the miR-188/ELF2 axis [17]. Moreover, miR-34c-5p inhibited the tumorigenesis of NPC [18]. Nevertheless, the association between circCRIM1 and miR-34c-5p remains largely unknown.

FOS-like antigen 1 (FOSL1) is a member of the AP-1 family, which is closely associated with cellular processes (cell proliferation, etc.) [19, 20]. In addition, FOSL1 is involved in the progression of malignant tumours (oesophageal cancer, lung cancer, etc.) [21, 22]. However, the relationship among circCRIM1, miR-34c-5p and FOSL1 in NPC needs to be explored.

Based on this background, this research focused on the function of circCRIM1 in NPC and the relationship among circCRIM1, miR-34c-5p and FOSL1. We hope that the results of our study will provide a new method for NPC treatment.

## Materials and methods

### Cell culture

NP69 and human nasopharyngeal carcinoma cells (SUNE-1, CNE2, C666-1, CNE-1, HNE1 and 5-8F) were purchased from the American Type Culture Collection (ATCC) and maintained in DMEM (Thermo Fisher Scientific) containing 10% FBS, penicillin and streptomycin (1%, Thermo Fisher Scientific) at 37 °C and 5% CO_2_.

### Cell transfection

The shRNA-directed target CRIM1 (sh-CRIM1) and negative control (sh-NC) were generated by GenePharma (Shanghai, China). Sh-CRIM1 and sh-NC were transfected into NPC cells. After incubation, the supernatant was collected by centrifugation. Then, the supernatants were filtered, and the cells were infected with particles for 48 h. Puromycin (2.5 μg/mL, Sigma, MO, USA) was applied to select the cells.

NPC cells were transfected with the miR-34c-5p mimics/inhibitor, pcDNA3.1-FOSL1 or NC (NC mimics/inhibitor or pcDNA3.1) using Lipofectamine 3000. The miR-34c-5p mimics/inhibitor, pcDNA3.1-FOSL1 and NC were purchased from GenePharma.

### MTT assay

NPC cells (5 × 10^3^ cells/well) were treated with the NC or sh-CRIM1 for 12, 24, 48 or 72 h. After that, NPC cells were treated with MTT solution (20 μL) for 2 h. The absorbance (490 nm) was determined by a microplate reader. The protocol was performed as previously described [23].

### Western blot assay

Cells were lysed in RIPA buffer (Beyotime). Equal amounts (20 µg) of protein from each group were separated by SDS–PAGE, and the proteins were then transferred onto a PVDF membrane (Beyotime). Then, the membrane was blocked and incubated with primary antibodies against FOSL1 (1:1000) and GAPDH (1:1000) and the corresponding secondary antibody (1:5000). All antibodies were purchased from Abcam (MA, USA). GAPDH was used for normalization. The protocol was performed as described previously [24].

### qRT–PCR

Total RNA was obtained with TRIzol (TaKaRa). Subsequently, cDNA was synthesized with a reverse transcription kit (TaKaRa). The protocol was as follows: 94 °C for 2 min, followed by 35 cycles of 94 °C for 30 s and 55 °C for 45 s. The primers were designed by GenePharma, and the data were quantified by the 2^−ΔΔCt^ method. β-actin or U6 was used for normalization. The following primers were designed by GenePharma: circCRIM1, forward, 5ʹ-GCCTTTCCCTGCTACTTGTG-3ʹ and reverse 5ʹ-AGAGCTTCCAAAGGCTAGGG-3ʹ; miR-34c-5p, forward, 5ʹ -GCCGCAGTGCAATGATGAAA-3ʹ and reverse 5ʹ -GTCGTATCCAGTGCAGGGTCCGAGGTATTCGCACTGGATACGACATGCC-3ʹ; FOSL1, forward, 5ʹ-GCCGCAGTGCAATGATGAAA-3ʹ and reverse 5ʹ-GTCGTATCCAGTGCAGGGTCCGAGGTATTCGCACTGGATACGACATGCC-3ʹ; β-actin, forward, 5ʹ-CCAGGTGGTCTCCTCTGA-3ʹ and reverse 5ʹ-GCTGTAGCCAAATCGTTGT-3ʹ; and U6, forward, 5ʹ-CTCGCTTCGGCAGCACA-3ʹ and reverse 5ʹ-AACGCTTCACGAATTTGCGT-3ʹ.

### Cell migration and invasion assays

The upper chamber was pretreated with Matrigel (100 μL, not included in the migration assay). NPC cells (1.0 × 10^6^ cells per chamber) in medium (1% FBS) were seeded into the upper chamber. Meanwhile, RPMI 1640 medium containing 10% FBS was added to the lower chamber. Subsequently, the cells in the chamber were rinsed and fixed with 5% glutaraldehyde at 4 °C. Then, the cells were stained with crystal violet (0.1%) for 20 min. The cells were observed under a microscope after the chamber was washed.

### Dual luciferase reporter assay

CircCRIM1 and FOSL1 (3ʹ-UTR) sequences with miR-34c-5p binding sites were purchased from GenePharma and cloned into the psiCHECK2 vectors (Promega) to establish circCRIM1 (WT/MUT) and FOSL1 (WT/MUT). NPC cells containing NC/ miR-34c-5p mimics were treated with circCRIM1 (WT/MUT) or FOSL1 (WT/MUT). The data were acquired by the luciferase system (Promega). The protocol was performed as previously described [25].

### Statistical analysis

Three independent experiments were performed for each group. In addition, all data are expressed as the mean ± standard deviation (SD). Differences were analysed by Student’s *t* test (only 2 groups) or one-way analysis of variance (ANOVA) followed by Tukey’s test (more than 2 groups). GraphPad Prism (version 7) was used for statistical analysis. *P* < 0.05 indicated statistically significant differences.

## Results

### CircCRIM1 and FOSL1 were upregulated in NPC cells, while miR-34c-5p was downregulated

**Fig. 1:**
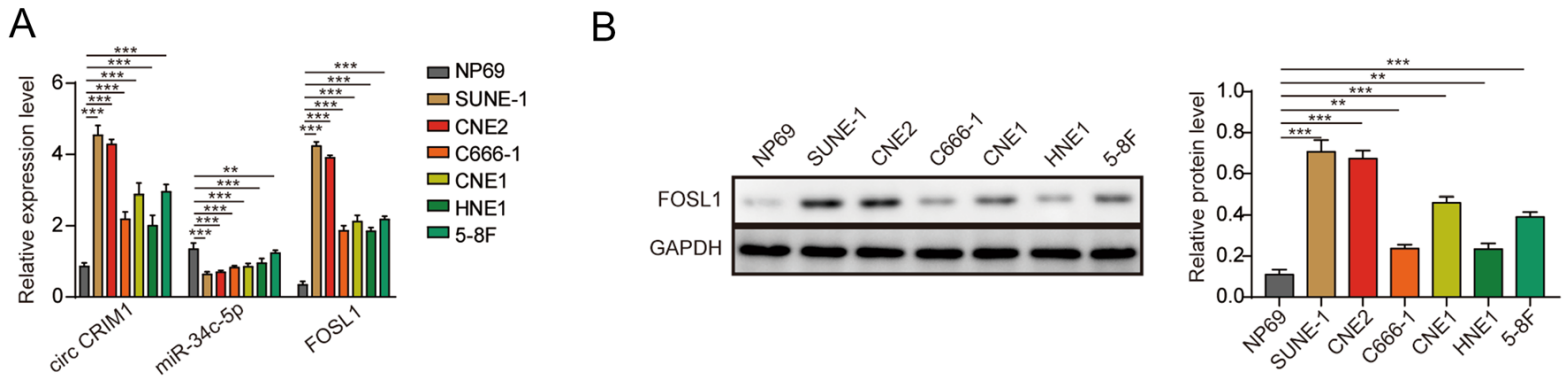
CircCRIM1 and FOSL1 were upregulated in NPC cells, while miR-34c-5p was downregulated. **A** The CircCRIM1, miR-34c-5p and FOSL1 levels in NP69, SUNE-1, CNE2, C666-1, CNE-1, HNE1 and 5-8F cells were determined by RT–qPCR. **B** The FOSL1 levels in NP69, SUNE-1, CNE2, C666-1, CNE-1, HNE1 and 5-8F cells were determined by Western blot. GAPDH was used for normalization. ^**^P < 0.01 compared with NP69. All data are expressed as the mean ± standard deviation (SD)

To elucidate the role of circCRIM1, miR-34c-5p and FOSL1 in NPC, RT–qPCR was performed. As revealed in Fig. 1A–C, the expression levels of circCRIM1 and FOSL1 in NPC cells were significantly higher than those in NP69 cells. The miR-34c-5p level in NPC cells was notably lower than that in NP69 cells (Fig. 1A). Since the levels of circCRIM1, miR-34c-5p and FOSL1 were changed most significantly in SUNE-1 and CNE2 cells, these two cell lines were selected for use in subsequent experiments. Taken together, these results demonstrated that circCRIM1 and FOSL1 were upregulated in NPC cells, while miR-34c-5p was downregulated.

### Silencing circCRIM1 significantly inhibited the proliferation, migration and invasion of NPC cells

**Fig. 2:**
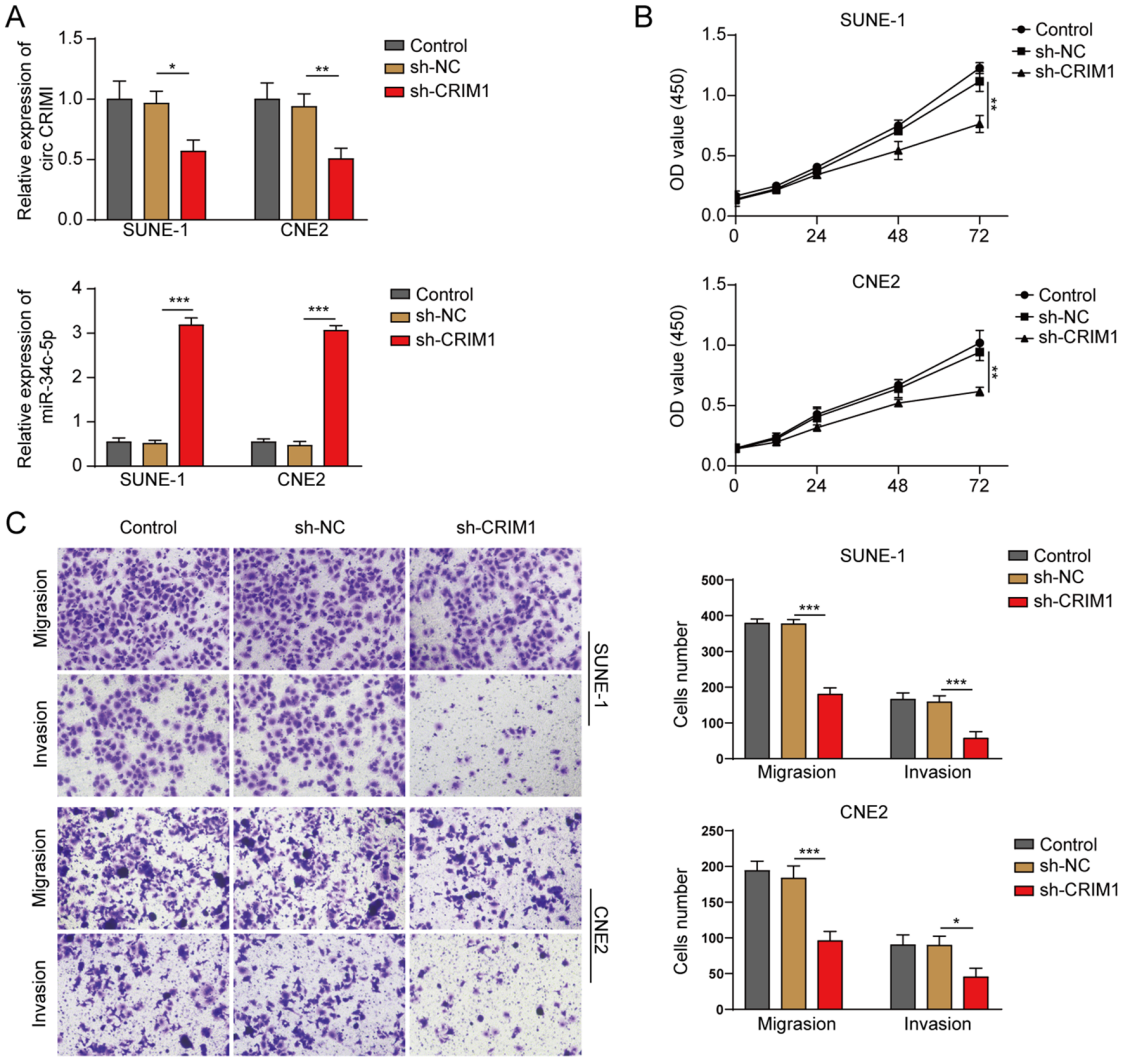
Silencing circCRIM1 significantly inhibited NPC cell invasion, proliferation and migration. SUNE-1 or CNE2 cells were transfected with sh-NC or sh-circCRIM1. Then, **A** the circCRIM1 and miR-34c-5p levels in NPC cells were determined by RT–qPCR. **B** NPC cell viability was assessed by the MTT assay. **C** NPC cell invasion and migration were assessed by the transwell assay. ^**^P < 0.01. All data are expressed as the mean ± standard deviation (SD)

To assess the function of circCRIM1 in NPC, NPC cells were transfected with sh-CRIM1. Then, the efficiency was determined by RT-qPCR. The circCRIM1 levels in NPC cells were significantly decreased by sh-CRIM1, and the expression of miR-34c-5p in NPC cells was notably upregulated by circCRIM1 shRNA (Fig. 2A). In addition, circCRIM1 shRNA attenuated the viability of NPC cells (Fig. 2B). Moreover, silencing circCRIM1 notably suppressed the invasion and migration of NPC cells (Fig. 2C). Altogether, these results demonstrated that silencing circCRIM1 attenuated the migration, proliferation and invasion of NPC cells.

### Overexpression of miR-34c-5p alleviated the invasion, proliferation and migration of NPC cells

**Fig. 3:**
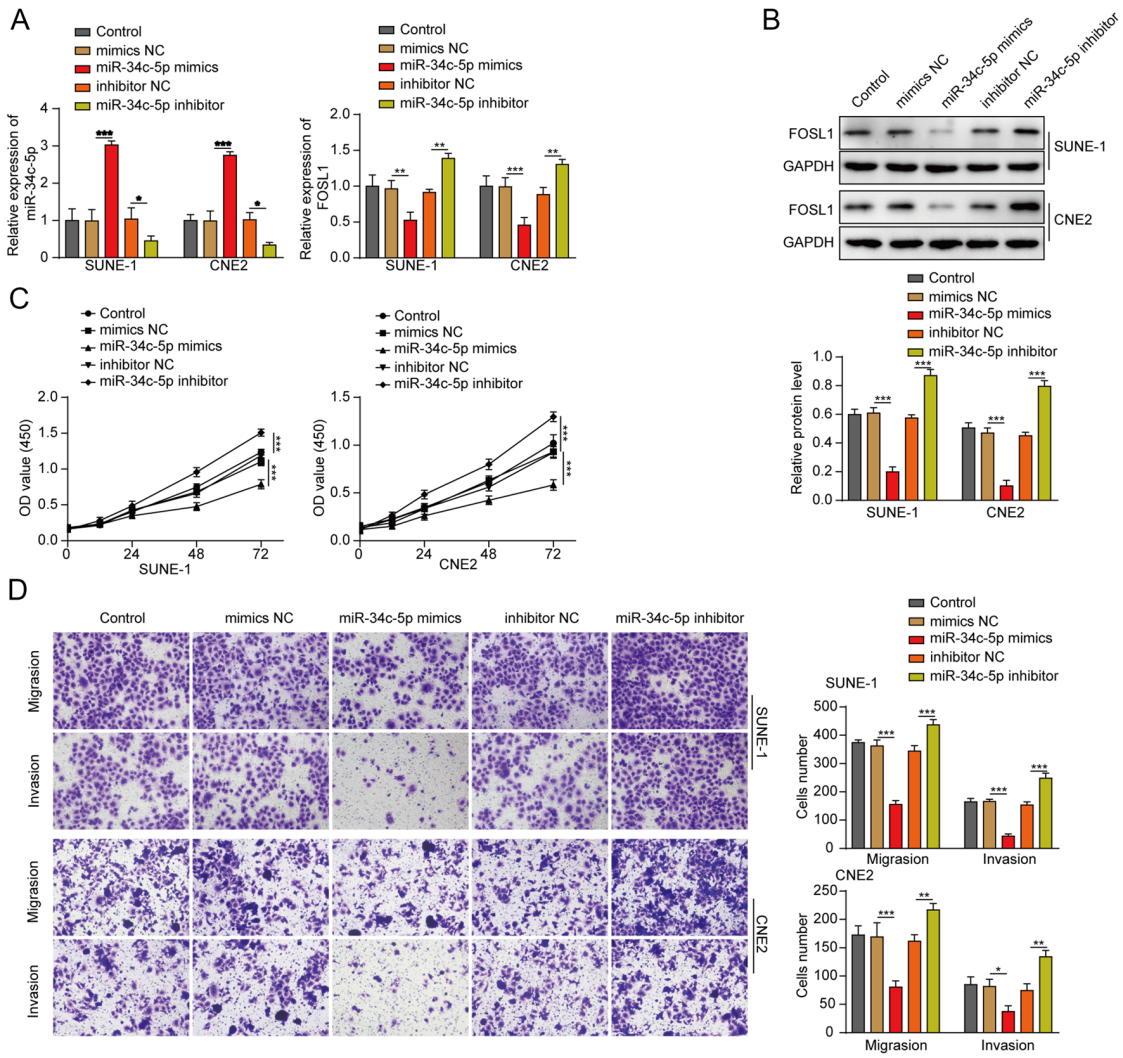
MiR-34c-5p upregulation significantly attenuated NPC cell invasion, proliferation and migration. SUNE-1 or CNE2 cells were treated with the miR-34c-5p mimics/inhibitor or NC. **A** The MiR-34c-5p levels in NPC cells were determined by RT–qPCR. **B** The FOSL1 levels in NPC cells were determined by Western blot. GAPDH was used for normalization. **C** NPC cell viability was assessed by the MTT assay. **D** NPC cell invasion and migration were assessed by the transwell assay. ^**^P < 0.01. All data are expressed as the mean ± standard deviation (SD)

To assess the effect of miR-34c-5p on NPC cell growth, NPC cells were transfected with miR-34c-5p mimics/inhibitor. As shown in Fig. 3A, the level of miR-34c-5p in NPC cells was notably upregulated by miR-34c-5p but decreased by the miR-34c-5p inhibitor. Moreover, the expression of FOSL1 in cells was markedly upregulated by the miR-34c-5p inhibitor, while miR-34c-5p mimics obviously inhibited FOSL1 levels (Fig. 3B). Additionally, miR-34c-5p upregulation notably inhibited the viability of NPC cells, while miR-34c-5p downregulation exerted the opposite effect (Fig. 3C). Moreover, NPC cell invasion and migration were significantly decreased by miR-34c-5p mimics but increased by miR-34c-5p depletion (Fig. 3D). In summary, overexpression of miR-34c-5p significantly inhibited the invasion, proliferation and migration of NPC cells.

### MiR-34c-5p was shown to bind circCRIM1 and FOSL1 in NPC cells

**Fig. 4:**
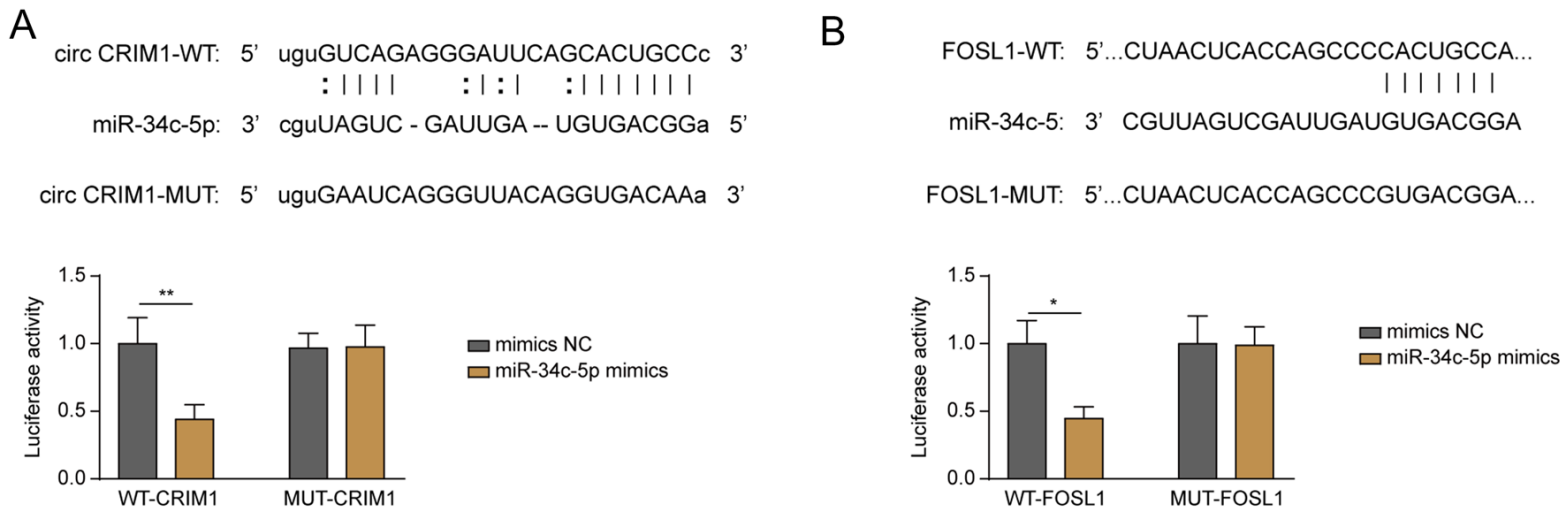
MiR-34c-5p was shown to bind circCRIM1 and FOSL1 in NPC cells. **A** The binding sites between circCRIM1 and miR-34c-5p are shown. The luciferase activity of WT/MUT-circCRIM1 was determined by the dual luciferase assay. **B** The binding sites between miR-34c-5p and FOSL1 are shown. The luciferase activity of WT/MUT-FOSL1 was determined by the dual luciferase assay. ^**^P < 0.05. All data are expressed as the mean ± standard deviation (SD)

To assess the relationship between miR-34c-5p and circCRIM1 in NPC cells, the dual luciferase assay was performed. As shown in Fig. 4A, circCRIM1 had putative miR-34c-5p binding sites, and the luciferase activity of WT-circCRIM1 was significantly downregulated by miR-34c-5p upregulation. However, miR-34c-5p had a very limited effect on the luciferase activity of MUT-circCRIM1 (Fig. 4A). Moreover, FOSL1 was predicted to be the downstream target of miR-34c-5p, and miR-34c-5p mimics notably inhibited the luciferase activity of WT-FOSL1 (Fig. 4B). Taken together, these results showed that miR-34c-5p could bind with circCRIM1 and FOSL1 in NPC.

### FOSL1 upregulation reversed the circCRIM1 shRNA-induced inhibition of NPC cell invasion, proliferation and migration

**Fig. 5:**
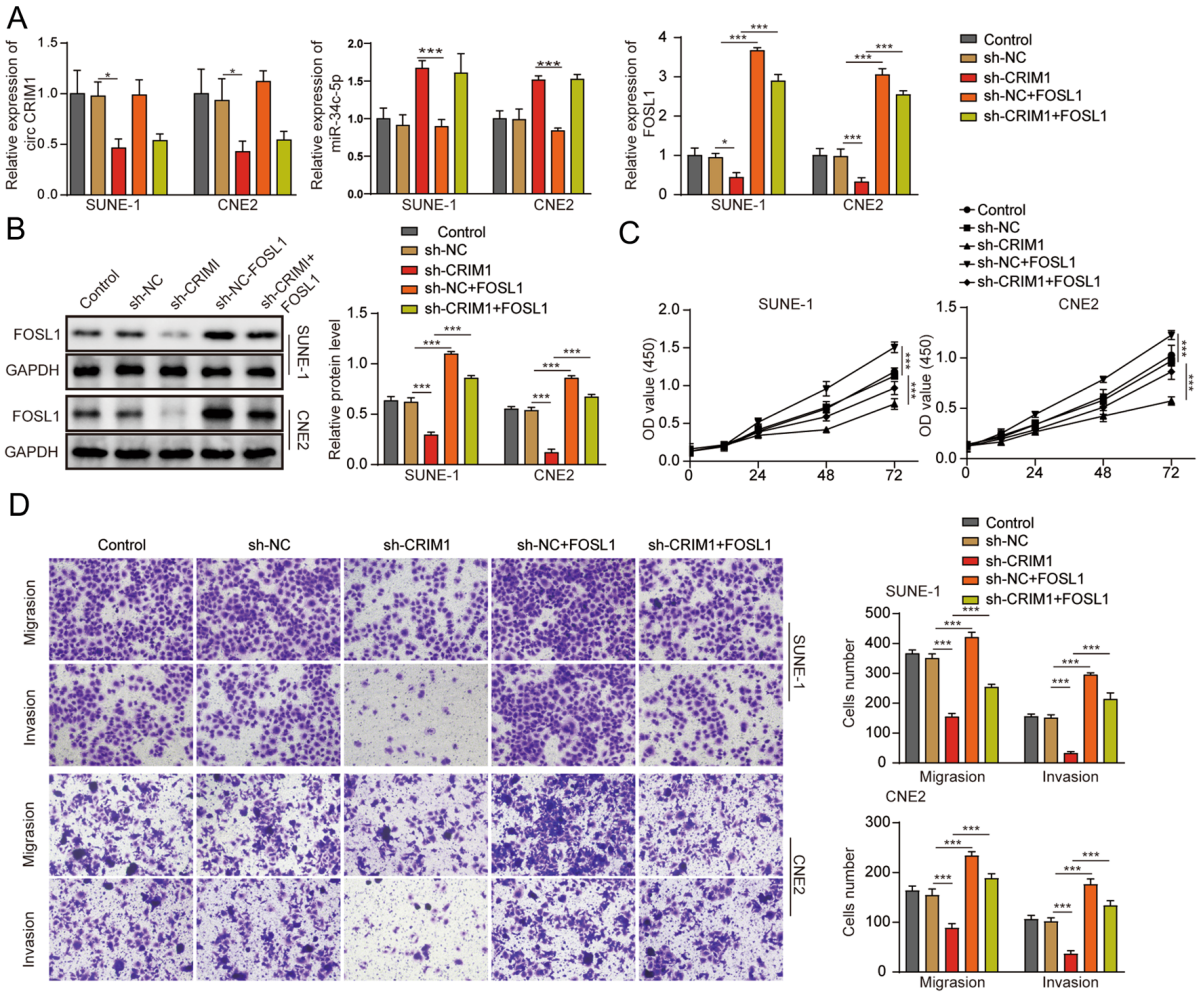
Overexpression of FOSL1 reversed the circCRIM1 shRNA-induced inhibition of NPC cell invasion, proliferation and migration. SUNE-1 or CNE2 cells were treated with the control, sh-NC, sh-circCRIM1, sh-NC + FOSL1 or sh-circCRIM1 + FOSL1. Then, **A** the circCRIM1, miR-34c-p and FOSL1 levels in NPC cells were determined by RT–qPCR. **B** The FOSL1 levels in NPC cells were determined by Western blot. GAPDH was used for normalization. **C** NPC cell viability was assessed by the MTT assay. **D** NPC cell invasion and migration were assessed by the transwell assay. ^**^P < 0.01. All data are expressed as the mean ± standard deviation (SD)

To elucidate the role of FOSL1 in circCRIM1 shRNA-mediated NPC progression, NPC cells were transfected with pcDNA3.1-FOSL1. As shown in Fig. 5A and B, the FOSL1 levels in NPC cells were decreased by circCRIM1 shRNA, while the effect of sh-CRIM1 on FOSL1 expression was restored by FOSL1 overexpression. In addition, the circCRIM1 shRNA-induced decrease in NPC cell viability was significantly abolished by FOSL1 (Fig. 5C). Consistently, the migration and invasion of circCRIM1 shRNA-treated NPC cells were obviously increased by the upregulation of FOSL1 (Fig. 5D). In summary, overexpression of FOSL1 reversed the circCRIM1 shRNA-induced downregulation of NPC cell invasion, proliferation and migration.

## Discussion

NPC treatments often fail due to NPC metastasis [26, 27]. In this study, circCRIM1 was found to be upregulated in NPC cells, and silencing circCRIM1 attenuated the invasion, proliferation and migration of NPC cells. CircCRIM1 knockdown can reportedly alleviate the tumorigenesis of NPC [12]. In addition, our research suggested that circCRIM1 binds with miR-34c-5p in NPC cells, and FOSL1 was identified to be downstream of miR-34c-5p in NPC cells. Therefore, our study first explored the relationship among circCRIM1, miR-34c-5p and FOSL1 in NPC cells.

It has been reported that circRNAs can regulate cellular processes, enabling them to thereby regulate the progression of multiple diseases, including cancers [28, 29]. Previous studies have indicated that circCRIM1 plays a role in malignant tumours. For example, silencing circCRIM1 disrupted osteosarcoma cell invasion, proliferation and migration via mediation of the miR-432-5p/HDAC4 axis [30]. Our research suggested an association between circCRIM1 and the miR-34c-5p/FOSL1 axis, which further supports its role in NPC. MiR-34c-5p is a key inhibitor of cancer progression [31, 32]. Previous studies suggested that silencing circCRIM1 attenuates the progression of NPC via its binding to miR-34c-5p. Moreover, Hong et al. suggested that FOXQ1 is upregulated by circCRIM1 in NPC [12], in contrast to our finding. FOXQ1 was reported to promote cell growth [33]. Thus, the similar functions of FOSL1 and FOXQ1 might account for the various functions of circCRIM1 in NPC. The above results suggest that circCRIM1 promotes NPC.

A previous study demonstrated that c-Myc and Notch1 are potentially direct targets of miR-34c-5p [10, 18]. FOSL1 reportedly participates in cancer tumorigenesis. For instance, FOSL1 upregulation led to the tumorigenesis of head and neck squamous cell carcinoma [34], and FOSL1 promoted the proliferation of colorectal cancer cells [35]. Consistently, the results of our study further suggest that FOSL1 is the target of miR-34c-5p in NPC cells, which furthers our understanding of the mechanism underlying miR-34c-5p in NPC. Moreover, a previous study indicated that EGFR-PKM2 signalling promotes the malignant behaviour of NPC cells via the inactivation of FOSL1 and ANTXR2 [36], consistent with our results herein. EGFR is known to be the key promoter of multiple cancers (e.g. NSCLC, breast cancer [37–39]), and FOSL1 has been confirmed to be indirectly targeted by circCRIM1. Thus, circCRIM1 might act as the promoter of the EGFR pathway, and we plan to investigate the relationship between circCRIM1 and the EGFR pathway in the future.

Indeed, this study does have the following limitations: (1) more miRNAs downstream of circCRIM1 in NPC need to be explored, and (2) additional targets of miR-34c-5p in NPC remain unexplored. Thereafter, more analysis is necessary in the future.

## Conclusion

This research was limited by the lack of in vivo experiments, which are needed to further validate the results. Thus, more investigations are needed in the future.

In conclusion, silencing circCRIM1 significantly attenuated the development of NPC through the miR-34c-5p/FOSL1 axis. Therefore, our study might shed new light on exploring new strategies for NPC treatment.

## Data Availability

Not applicable.

## Abbreviations

circCRIM1: circRNA cysteine-rich transmembrane bone morphogenetic protein regulator 1
FOSL1: Fos-like antigen-1
NPC: Nasopharyngeal carcinoma
AP-1: Transcription factor activator protein-1
